# Association Between Hospital-Based Addiction Consult Service Implementation and Hospital Length-of-Stay and 30-Day Readmission Rates for Patients with Opioid Use Disorder

**DOI:** 10.1007/s11606-026-10565-6

**Published:** 2026-06-29

**Authors:** Margaret Lowenstein, Seiyoun Kim, Suzy Landon, Lin Xu, David S. Mandell, Jeanmarie Perrone, Paula Chatterjee

**Affiliations:** 1 Perelman School of Medicine at the University of Pennsylvania, 3600 Civic Center Blvd, 7th Floor, Philadelphia, PA 19104 USA; 2 University of Pennsylvania Center for Addiction Medicine and Policy, Philadelphia, PA USA; 3 Leonard Davis Institute of Health Economics, Philadelphia, PA USA

**Keywords:** addiction consult services, hospital-based addiction care, opioid use disorder, medication for opioid use disorder, quality of care

## Abstract

**Background:**

Addiction consult services (ACSs) are a growing hospital-based care model that increases quality of care for patients with substance use disorders (SUDs). One implementation barrier has been concern about negative financial impacts for health systems.

**Objective:**

To examine whether starting an ACS changes hospital length-of-stay and 30-day readmissions for patients with opioid use disorder (OUD) served in a large academic health system.

**Design:**

Quasi-experimental difference-in-differences study of opioid-related hospital admissions from January 2018 to December 2024, comparing one hospital that implemented an ACS to three hospitals in the same urban, academic system in Philadelphia, PA without ACSs.

**Participants:**

Adults (≥ 18 years) with opioid-related hospitalizations.

**Intervention:**

A fully staffed, hospital-based, multidisciplinary ACS launched in July 2023.

**Main Measures:**

Primary outcomes were hospital length-of-stay and 30-day readmissions. Secondary outcomes were receipt of any medication for opioid use disorder (MOUD) during hospitalization, discharge on a therapeutic MOUD dose, emergency department visits within 6 months of discharge, and discharges before medically advised.

**Key Results:**

In unadjusted analyses, ACS implementation was associated with a 5 percentage point increase in MOUD receipt (95% CI 0–10) and a 9 percentage point increase in discharge on therapeutic MOUD (95% CI 5–13), without significant changes in length-of-stay or 30-day readmissions. In adjusted analyses, therapeutic MOUD at discharge increased by 8 percentage points (95% CI 4–12), with no significant differences in length-of-stay or 30-day readmission. Results were robust to sensitivity analyses with alternative comparison groups and after accounting for the COVID-19 pandemic.

**Conclusions:**

Implementation of an ACS improved evidence-based care for hospitalized patients with OUD without prolonging length-of-stay or increasing readmissions.

**Graphical Abstract:**

**Figure:**
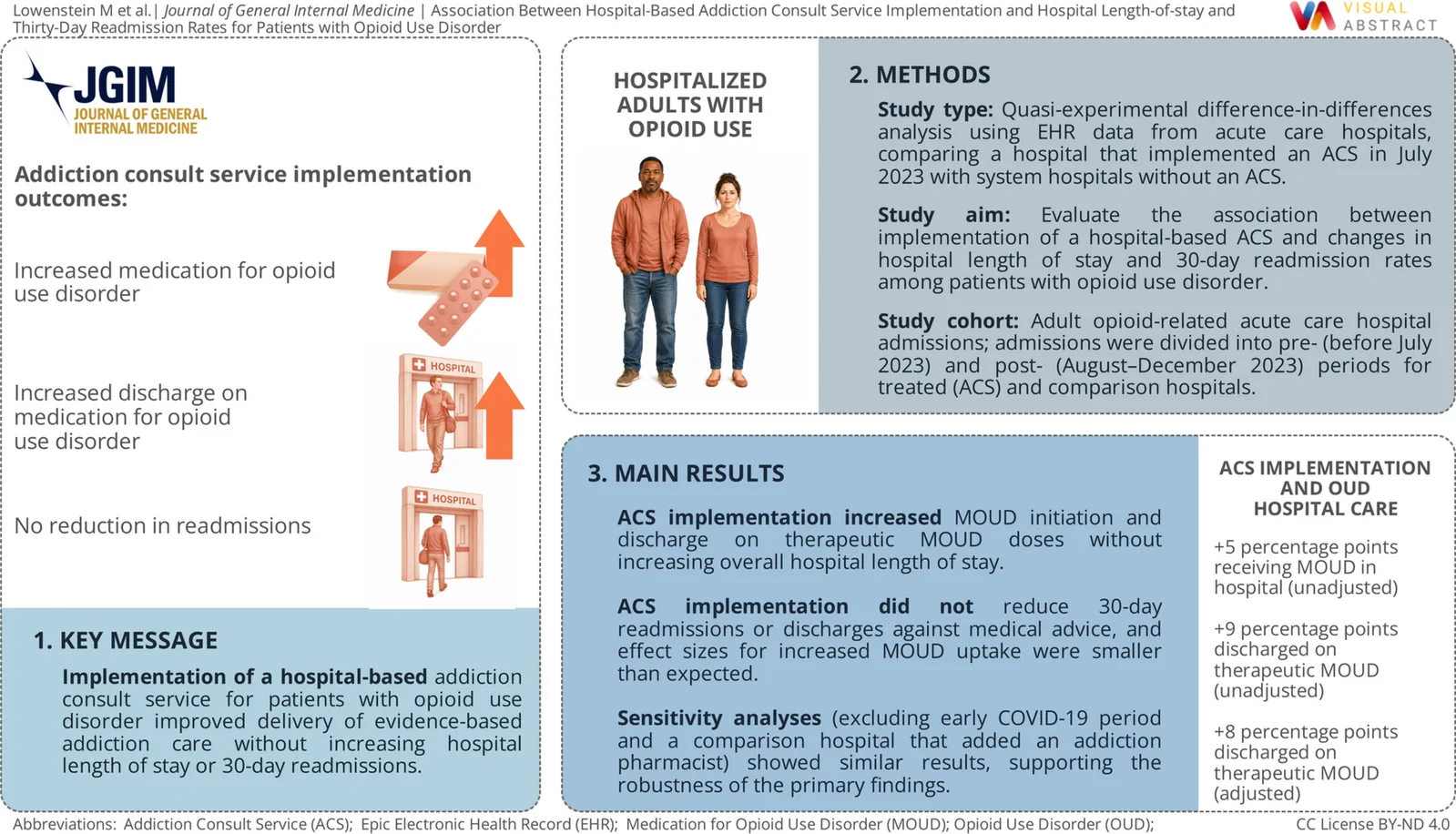

**Supplementary Information:**

The online version contains supplementary material available at https://doi.org/10.1007/s11606-026-10565-6.

## INTRODUCTION

The rise of opioid use disorder (OUD) and its complications over the last several decades has led to high morbidity and mortality for affected patients and high costs to health systems.^1–5^ Hospitals are increasingly recognized as critical touchpoints for engaging individuals with OUD in evidence-based treatment and harm reduction interventions.^6^ Initiation of medications for OUD (MOUD), such as buprenorphine and methadone, substantially reduces all-cause and overdose mortality,^7^ and starting MOUD in an acute care setting increases engagement in care following discharge.^8,9^

Addiction consult services (ACSs) are a growing model aimed at improving care for hospitalized patients with OUD and other substance use disorders (SUDs). While models vary, ACSs are typically multidisciplinary teams (including physicians, social workers, pharmacists, and peer recovery professionals, for example) that focus on acute management of withdrawal and pain, MOUD initiation, discharge planning, and linkage to ongoing care.^10^ Several retrospective cohort studies and two recent randomized controlled trials have demonstrated that ACS implementation is associated with increased rates of MOUD initiation and treatment linkage after discharge.^11–13^

Despite these benefits, health system leaders nationally have expressed concerns that ACSs may increase utilization or costs under existing payment incentives. For example, along with the cost of the ACS team itself, which may not be fully offset by billing,^14,15^ there are concerns that hospital length-of-stay could increase after ACS implementation due to the time required to introduce and titrate MOUD to maximum efficacy. Readmissions could increase due to challenging care transitions post-discharge, or because ACSs increase trust and engagement with health care institutions in ways that promote care seeking. These dynamics are further complicated by the insurance demographics of individuals with SUD, who are often Medicaid-enrolled or uninsured.^16,17^ For these reasons, offering a financial or “business case” for ACSs has been cited as an important implementation strategy.^17,18^

Rigorous evidence to support this case has been mixed, descriptive, and limited to pre-post or retrospective cohort designs.^12^ In this study, we use a difference-in-differences approach to examine the causal relationship between the implementation of an ACS and changes in hospital length-of-stay and 30-day readmission rates for patients with OUD across a large academic health system. We also assess outcomes of MOUD initiation, discharge on therapeutic MOUD dose, 6-month emergency department (ED) visits, and discharges before medically advised following ACS implementation to further characterize spillover effects of ACSs on care and utilization for patients with OUD.

## METHODS

### Study Setting and Consult Intervention

This study was conducted at a large academic health system in Philadelphia, PA, which has one of the highest overdose death rates among US cities.^19^ The study period spanned January 1, 2018–December 31, 2024, and included four urban hospitals in the same health system: a large tertiary referral hospital, a level I trauma center, and two hospitals with associated psychiatric crisis centers.

One of the four hospitals implemented an ACS pilot in April 2023 and a fully scaled model in July 2023, covering Monday–Friday 8 am to 5 pm. The ACS team included an attending physician with board certification in addiction medicine, an addiction medicine fellow, a dedicated hospital social worker with expertise in substance use care, and a dedicated peer recovery specialist. The team also included rotating medical student, resident, and fellow trainees. The ACS team provided clinical recommendations for admitted patients on medical-surgical floors at the request of primary hospital teams, and primary teams implemented ACS treatment recommendations. The ACS social worker and peer specialist further supported patients with discharge planning, psychosocial support, harm reduction education, and access to social services to address unmet needs such as clothing, transportation, and housing resources at discharge. Although ACSs typically care for a range of SUDs, in this study, we examined outcomes for patients with OUD because this was the most common condition managed by the institution's ACS team.

### Study Population

The admission-level analysis included acute care hospital admissions between January 1, 2018, and December 31, 2024. We included admissions for patients ≥ 18 with an International Classification of Disease (ICD)−10 code consistent with opioid use during index admission or in another encounter within the prior year (Appendix Table 1). Hospitalizations could be primarily for the opioid-related diagnosis or another cause.

### Data Source

We used data from the Epic electronic health record via the Clarity reporting database (Hyperspace 2025 ; Epic Systems Corporation). We extracted patient demographic characteristics including age, sex, race, ethnicity, and insurance status as well as data on index hospital visits for inpatient and observation admissions including comorbidities, medications provided, level of care, consults, length-of-stay, and acute care utilization before and after the admission.

### Outcomes

#### Primary Outcomes

Our primary outcomes were hospital length-of-stay and 30-day hospital readmission. We chose these outcomes because hospitals have clinical, operational, and financial incentives to reduce length-of-stay and 30-day readmissions, and both outcomes are salient to hospital administrators.^20–25^ Hospital length-of-stay was defined as the number of days between admission and discharge dates. Thirty-day readmission was defined as readmission to any hospital in the health system within 30 days of discharge.

#### Secondary Outcomes

We had four secondary outcomes specifically related to the quality of care for patients with OUD: (1) MOUD receipt, (2) discharge on a therapeutic MOUD dose, (3) proportion of patients with an ED visit within 6 months of discharge within the study health system, and (4) discharges before medically advised.

MOUD receipt was defined as administration of buprenorphine or methadone, or both, during the hospitalization. Discharge on a therapeutic MOUD dose was defined as receiving a buprenorphine discharge prescription or a methadone dose of ≥ 60 mg administered within the final 24 h of hospitalization. Methadone doses of ≥ 60 mg are considered the minimum therapeutic dose for initial stabilization, although many patients are ultimately titrated to higher maintenance doses.^26^ Since methadone is not prescribed at discharge due to federal regulations requiring opioid treatment programs to administer outpatient methadone treatment for OUD,^27^ we used methadone doses ≥ 60 mg in the last 24 h of hospitalization as a proxy for discharge on a therapeutic dose. Buprenorphine can be rapidly titrated to therapeutic doses,^26^ and 98% of buprenorphine discharge doses among our cohort exceeded a minimum therapeutic range of ≥ 8 mg; for this reason, we did not distinguish between subtherapeutic and therapeutic buprenorphine doses in this sample. Finally, discharges before medically advised were defined as any discharge where “left against medical advice” was the discharge disposition.^28–32^

### Data Analysis

#### Sample Characteristics

Baseline patient characteristics were calculated for 2022, the year preceding ACS implementation. We compared patients admitted to the hospital that implemented an ACS with those admitted to the other three hospitals without an ACS. Patient characteristics included age, sex, race, insurance type, Charlson Comorbidity Index (CCI) score (weighted comorbidity measure of multiple clinical conditions and their association with mortality),^33^ and presence of serious injection-related infection during the hospitalization. *P*-values were obtained using *t*-tests for continuous variables and Pearson’s chi-squared tests for categorical variables.

#### Unadjusted Trends in Primary and Secondary Outcomes

We plotted monthly unadjusted trends in the outcomes from 2018 to 2024 for patients admitted to the hospital that implemented an ACS vs. those admitted to hospitals that did not implement an ACS, to observe changes in trends before and after ACS implementation.

#### Difference-in-Differences Regressions

We used a difference-in-differences approach to estimate the association between ACS implementation and changes in outcomes. This approach compares changes in outcomes before and after July 2023 (which was the time of ACS implementation) at the hospital that implemented an ACS relative to control hospitals within the same health system without an ACS. Under key assumptions, this approach accounts for background trends and potential unobserved confounders that may have impacted the outcomes over time.^34^ One assumption is that the ACS and non-ACS hospital sites should be subject to “common shocks,” meaning that other than the exposure being tested (i.e., ACS implementation), outside events during the study period should impact the intervention and control groups equally.^34^ In this case, all hospitals in the sample were subject to the same health system policies and guidelines and the same drug supply changes. Another assumption is that outcomes must follow parallel trends prior to the exposure—meaning that trends in outcomes must be similar in the pre-period. This can be transparently observed by plotting unadjusted trends over time, as described above.

The analysis compared changes in outcomes between patients with OUD admitted to the hospital with an ACS (“treated” admissions) and patients with OUD admitted to the other hospitals without ACS (“comparison” admissions), before and after the ACS was implemented in July 2023. We evaluated both unadjusted and adjusted models. The adjusted models controlled for age, sex, race, insurance type, CCI score, and the presence of serious injection-related infections. Standard errors were clustered at the admission level, and hospital and year fixed effects were included to account for time-invariant differences across hospitals and secular trends over time.

We conducted two sensitivity analyses. One of the non-ACS hospitals in the health system introduced limited addiction pharmacist support beginning in January 2021, with a full-time addiction pharmacist starting in July 2023 who worked with general medical and surgical teams. To account for potential contamination of the comparison group due to this pharmacy-based intervention, we conducted a sensitivity analysis excluding the hospital that implemented this intervention from the comparison group. Second, because our study period included the COVID-19 pandemic, we repeated analyses in which we censored data from March 2020 to December 2020 to account for disruptions caused by the initial phase of the pandemic and re-estimated the difference-in-differences models.

All analyses were conducted using Stata version 18.0 (StataCorp LLC). To account for multiple hypothesis testing with two primary outcomes, we applied a Bonferroni correction, setting the threshold for statistical significance at *P* < 0.03. This study was approved by the Institutional Review Board at the Perelman School of Medicine at the University of Pennsylvania.

## RESULTS

### Sample Characteristics

There were a total of 10,637 opioid-related admissions over the study period; 4390 in the treatment hospital and 6247 in the comparison hospitals. Following ACS implementation in the treatment hospital, 68% of admissions with an opioid-related diagnosis received an ACS consult.

We calculated baseline descriptive characteristics in 2022 (which was the year preceding ACS implementation). There were 1286 admissions for patients with OUD admitted to hospitals that did not implement an ACS (comparison group), and 661 admissions for patients with OUD treated at the hospital that implemented an ACS (treatment group, Table 1). Relative to the comparison group, admitted patients in the treatment group were older (mean age: 47.6 vs 45.1 years), more likely to be Black (47.5% vs 36.2%), and less likely to have a serious injection-related infection (9.7% vs. 12.9%) in the baseline year (2022).

**Table 1:** Sample Characteristics

		Comparison group	Treatment group
*N*		1286	661
Age, mean (SD)		45.1 (14.0)	47.6 (14.2)
Female, *n* (%)		536 (41.7%)	271 (41.0%)
Race, *n* (%)	White	716 (55.7%)	301 (45.5%)
	Black	465 (36.2%)	314 (47.5%)
	Other	105 (8.2%)	46 (7.0%)
Insurance type, *n* (%)	Medicaid	793 (61.7%)	393 (59.5%)
	Medicare	258 (20.1%)	157 (23.8%)
	Commercial	222 (17.3%)	105 (15.9%)
	Other	13 (1.0%)	6 (0.9%)
Charlson Comorbidity Index, mean (SD)		2.0 (2.7)	2.3 (3.0)
Serious injection-related infections, *n* (%)		166 (12.9%)	64 (9.7%)

Individuals in the comparison group reflected patients with an ICD-10 diagnosis corresponding to opioid-related disorder who were admitted to the three hospitals in the health system that did not implement an addiction consult service at any point during the study period. Individuals in the treatment group reflected patients with opioid-related disorder treated at the hospital that implemented an addiction consult service at any point during the study period

*SD*, standard deviation

### Unadjusted Trends in Primary and Secondary Outcomes

**Figure 1:**
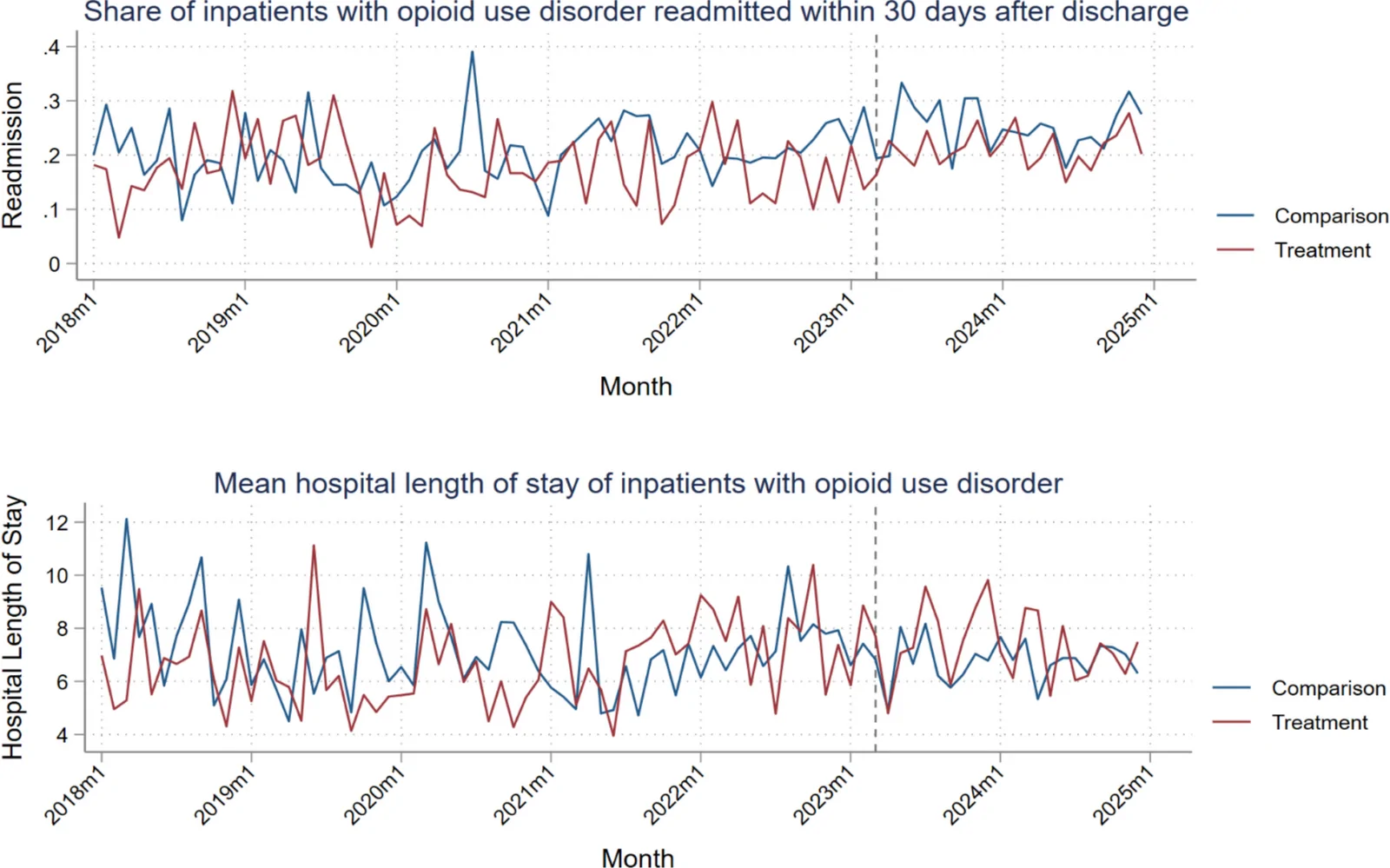
Unadjusted trends in primary outcomes. Individuals in the comparison group include patients with an ICD-10 diagnosis corresponding to opioid-related disorder who were admitted to the three hospitals in the health system that did not implement an addiction consult service at any point during the study period. Individuals in the treatment group reflected patients with opioid-related disorder treated at the hospital that implemented an addiction consult service at any point during the study period.

Figure 1 presents the unadjusted trends in the proportion of patients readmitted within 30 days of discharge and mean hospital length-of-stay among patients with OUD. Prior to ACS implementation, outcomes were similar between treatment and comparison groups, with no major changes observed post-implementation.

**Figure 2:**
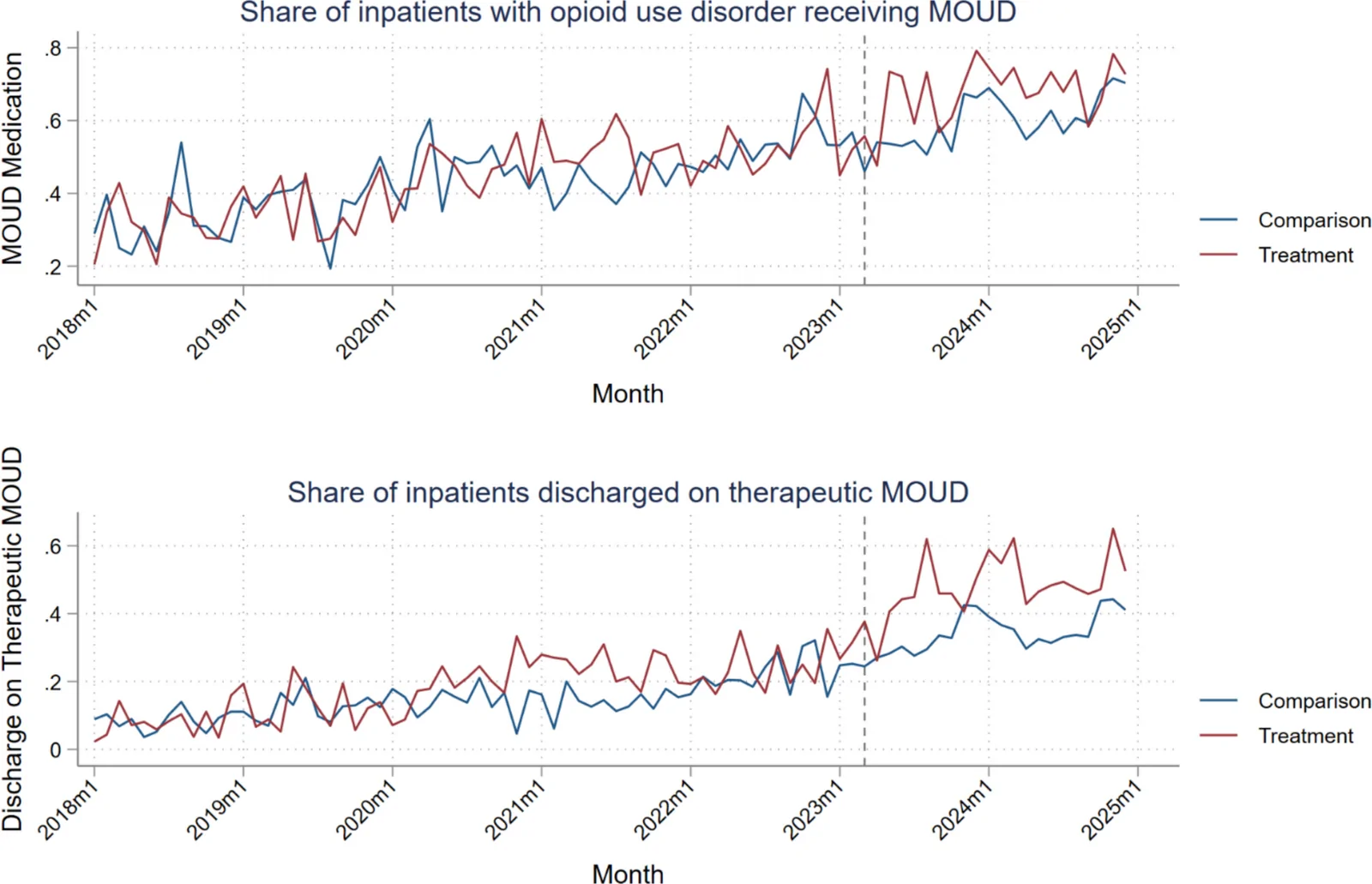
Unadjusted trends in secondary outcomes. Individuals in the comparison group include patients with an ICD-10 diagnosis corresponding to opioid-related disorder who were admitted to the three hospitals in the health system that did not implement an addiction consult service at any point during the study period. Individuals in the treatment group reflected patients with opioid-related disorder treated at the hospital that implemented an addiction consult service at any point during the study period. MOUD, medication for opioid use disorder.

Figure 2 presents unadjusted trends in the proportion of patients with OUD who received MOUD during hospitalization and the proportion of patients discharged on therapeutic MOUD doses. Prior to ACS implementation, the proportion of patients receiving any MOUD was similar in treatment and comparison hospitals (0.50 vs. 0.50), as was the proportion discharged on therapeutic MOUD (0.30 vs. 0.30). Following implementation, both proportions were higher in the hospital that implemented the ACS. The mean proportion receiving MOUD during hospitalization increased to 0.7 compared to 0.5 in the comparison group, and the proportion discharged on therapeutic MOUD rose to 0.4 compared to 0.3 in the comparison group. Differences between the treatment and comparison groups persisted over time, with higher proportions of MOUD receipt and therapeutic MOUD at discharge observed in the treatment group in most post-implementation months.

Unadjusted trends in the proportion of patients who were discharged before medically advised and proportion of patients with ED visits within 6 months of discharge were similar between groups before and after introduction of the ACS (Appendix Fig. 1). After adjusting for covariates, we observed similar patterns (Appendix Fig. 2).

### Difference-in-Differences (DiD) Regressions

Table 2 presents estimates from the unadjusted and adjusted difference-in-differences analyses for primary and secondary outcomes. There was no statistically significant association between ACS implementation and 30-day readmission rates (DiD estimate –1; 95% CI –5 to 3 percentage points) or mean hospital length-of-stay (DiD estimate 0.45 days; 95% CI –0.43 to 1.32 days). In unadjusted models, implementation of the ACS was associated with a statistically significant increase of 5 percentage points in the proportion of all patients with OUD receiving MOUD (95% CI 0 to 10 percentage points), and a 9 percentage point increase in the proportion of patients discharged on therapeutic MOUD (95% CI 5 to 13 percentage points), relative to the change observed among patients in the comparison group during the same period. In adjusted models, implementation of the ACS was associated with a statistically significant increase of 8 percentage points in the proportion of patients discharged on therapeutic MOUD (95% CI 4 to 12 percentage points), relative to the comparison group. There were no statistically significant differences in the proportion of patients with an ED visit in the 6 months following discharge or in the proportion of patients discharged before medically advised discharge between the treatment and comparison groups following ACS implementation in both unadjusted and adjusted models.

**Table 2:** Difference-in-Differences Regression Estimates

	Unadjusted	Adjusted
Readmission within 30 days of discharge (percentage points)	−0.01 [−0.05, 0.03]	0 [−0.04, 0.04]
Length of stay (days)	0.45 [−0.43, 1.32]	0.38 [−0.46, 1.22]
MOUD receipt (percentage points)	0.05* [0.00, 0.10]	0.03 [−0.01, 0.08]
Discharge on therapeutic MOUD (percentage points)	0.09*** [0.05, 0.13]	0.08*** [0.04, 0.12]
Discharge before medically advised (percentage points)	0 [−0.04, 0.04]	−0.01 [−0.05, 0.03]
Emergency department visit within 6 months of discharge (number of visits)	0.02 [−0.03, 0.06]	0.03 [−0.01, 0.08]

Depicted are regression estimates that correspond to differential changes in outcomes between patients with OUD admitted to the hospital with an ACS (“treatment”) relative to patients with OUD admitted to the other hospitals without ACS (“comparison”), before and after the ACS was implemented in July 2023. All models include hospital- and year-fixed effects. Adjusted models additionally controlled for age, sex, race, insurance type, Charlson Comorbidity Index score, and the presence of serious injection-related infections. 95% confidence intervals are presented in brackets

*p-value < 0.03

***p-value < 0.001

The main findings were generally robust to exclusion of one of the comparison hospitals and the exclusion of the COVID-19 period (Appendix Tables 2 and 3). In the sensitivity analysis excluding one of the comparison hospitals that implemented pharmacist support for generalist teams, ACS implementation was associated with a statistically significant 8 percentage point increase in the proportion of patients receiving MOUD (95% CI 2 to 14), and a 12 percentage point increase in the proportion discharged on therapeutic MOUD (95% CI 8 to 16) in unadjusted models. In the adjusted model, there was a 5 percentage point increase in the proportion of patients receiving MOUD (95% CI 0 to 10) and a 10 percentage point increase in the proportion discharged on therapeutic MOUD (95% CI 6 to 14).

In sensitivity analyses excluding the early months of the COVID-19 pandemic, ACS implementation was associated with a statistically significant 5 percentage point increase in the proportion of patients receiving MOUD (95% CI 0 to 12), and a 9 percentage point increase in the proportion discharged on therapeutic MOUD doses (95% CI 5 to 14) in unadjusted models. In the adjusted model, there was an 8 percentage point increase in the proportion of patients discharged on therapeutic MOUD (95% CI 4 to 12).

## DISCUSSION

In this study of four hospitals in a large, academic health system, we find no evidence of increased length-of-stay or readmission rates associated with the implementation of an ACS for hospitalized patients with OUD. We do find significant differential improvements in MOUD receipt and discharge on therapeutic MOUD at sites where ACS was available relative to those where it was not. Together, these findings suggest that the quality-improving benefits of ACSs can be realized without compromising certain important dimensions of hospital throughput and financing.

While prior literature has consistently demonstrated a positive relationship between ACSs and MOUD initiation, there have been mixed results on the relationship with readmissions and length-of-stay.^12,35–38^ Our study builds on the literature through its use of a quasi-experimental approach that seeks to account for background trends and unobserved confounders to inform a causal relationship. Our sample’s 30-day readmission rates were high—approaching 25–30% across sites—but did not differ significantly by ACS implementation. High readmission rates are common among a population of patients with complex, acute medical conditions compounded by care fragmentation, barriers to accessing SUD treatment, and housing instability. In our study and others, it is important to recognize that average differences between groups may obscure heterogeneous effects. For example, in some cases, length-of-stay may have declined because the ACS results in more efficient discharge planning, while in others, it may have increased because the ACS results in patients receiving more treatment while hospitalized.^12^

Nonetheless, improvements in quality of care for patients with SUDs need not come at the expense of increased health care use and should offer some reassurance to health system leaders considering adoption of ACSs but are concerned about its potential financial effects. Although we did not see reductions in health care use, which might further the business case for the ACS, there is evidence that higher rates of MOUD initiation reduce acute care use in the long run.^35^ Prior research has estimated that SUDs generate significant costs to the health care system that likely could be offset with effective interventions to curb future health care use,^39^ and a recent economic simulation study estimated that ACSs and initiation of MOUD are likely to be cost-effective based on accepted thresholds.^40^  

In addition, improvements in quality of care with addiction consultation have larger impacts outside of hospital finances. Our findings add to the growing literature that ACSs improve delivery of evidence-based care for patients with OUD.^12^ ACSs provide expert guidance on SUD treatment and complications, as well as strategies to address clinical challenges and an evolving drug supply.^6,41,42^ Beyond these benefits, ACSs may also serve as a key intervention for trainee and staff education to reduce stigma, and, through the delivery of higher quality care, ultimately improve patient-centered outcomes.^43,44^ MOUD initiation and exposure have wide-ranging benefits for patients, including reductions in morbidity and mortality as well as improvements in quality of life.^7^ Evidence from both observational work and randomized clinical trials demonstrate that hospital-based initiation of MOUD substantially increases in-hospital treatment uptake, post-discharge linkage to ongoing care, and sustained engagement with care.^12,13^ Despite this well-established clinical benefit, efforts to expand hospital-based addiction care are often hampered by expectations to demonstrate a favorable business case—standards not always applied to other evidence-based clinical interventions—highlighting structural barriers that impede adoption even when patient-centered outcomes are clear and compelling.

Finally, ACSs may offer other benefits to health systems. Prior work has shown high levels of clinician moral distress and burnout related to addiction care in hospitalized patients,^45,46^ and ACSs can play a role mitigating these feelings to improve job satisfaction and staff retention. Further, given the high prevalence of addiction among hospitalized patients and the general population,^47^ ensuring high-quality care for this population is important to achieving goals of population health. Finally, addiction is a chronic disease, and benefits of ACSs may extend over longer time periods or beyond the hospital setting. Alternative outcomes, including qualitative measures of patient and primary provider team experiences with ACSs, 30-day mortality, or rates of outpatient engagement with care can fully inform the complement of benefits associated with ACSs.

This study has limitations. First, our findings come from a single, academic health system and may not be broadly generalizable. Second, while our analytic approach was designed to minimize the contribution of unobserved confounders, there may be residual confounding from patient or clinician characteristics. In addition, with our focus on health care use, we cannot consider additional costs or offsets such as increased care seeking, the costs of staffing the consult service, or financial offsets from billing revenue. We also report readmissions only within the study health system and do not account for changes in outside health care use. Finally, there may have been attenuation in the intervention due to crossover of trainees and staff within the four hospitals in the health system, which would have biased the estimated impact of the ACS toward the null.

In conclusion, this study demonstrates that implementation of an ACS can ensure evidence-based care for hospitalized patients with OUD without compromising readmission rates or length-of-stay. These findings should reassure health system leaders interested in providing high-quality care for patients with OUD while balancing the financial pressures of increasingly competitive hospital markets.

## Supplementary Information

Below is the link to the electronic supplementary material.

Supplementary file1 (DOCX 767 KB)

## Data Availability

The data that support the findings of this study are not publicly available due to privacy restrictions but are available from the corresponding author upon reasonable request and with appropriate institutional approvals.
