# Supplementary material for: Association Between Hospital-Based Addiction Consult Service Implementation and Hospital Length-of-Stay and 30-Day Readmission Rates for Patients with Opioid Use Disorder
Source: J Gen Intern Med. 2026 Jun 29;41(13):3709–18. doi: 10.1007/s11606-026-10565-6 (PMC13631137; doi:10.1007/s11606-026-10565-6)
Supplement: Supplementary file 1 [file 11606_2026_10565_MOESM1_ESM.docx]

**Supplemental Appendix**

Table 1: International Classification of Disease-10 codes to identify opioid use disorder

Figure 1: Unadjusted trends in number of emergency department visits and discharges before medically advised

Figure 2: Trends in outcomes adjusted for seasonality

Table 2: Sensitivity analysis with exclusion of a comparison hospital

Table 3: Sensitivity analysis with exclusion of March-December 2020 for COVID-19 pandemic

**Table 1: International Classification of Disease-10 codes to identify opioid-related disorders**

| **Category** | **ICD-10 Codes** | **Description** |
| --- | --- | --- |
| **Opioid Poisoning/Overdose** | T40.0X* | Poisoning by opium |
|  | T40.1X* | Poisoning by heroin |
|  | T40.2X* | Poisoning by other opioids (e.g., morphine, codeine) |
|  | T40.3X* | Poisoning by methadone |
|  | T40.4* | Poisoning by synthetic narcotics (e.g., fentanyl, tramadol) |
|  | T40.6* | Poisoning by other/unspecified narcotics |
| **Opioid-Related Disorders** | F11.1* | Opioid abuse (with/without complications) |
|  | F11.2* | Opioid dependence (with/without complications) |
|  | F11.9* | Opioid use, unspecified (with/without complications) |
|  |  |  |

**Figure 1: Unadjusted trends in number of emergency department visits within 6 months of discharge and discharges before medically advised**

Notes: Individuals in the comparison group include patients with an ICD-10 diagnosis corresponding to opioid use disorder who were admitted to the three hospitals in the health system that did not implement an addiction consult service at any point during the study period. Individuals in the treatment group reflected patients with opioid use disorder treated at the hospital that implemented an addiction consult service at any point during the study period.

**Figure 2: Trends in outcomes adjusted for covariates**

Notes: Individuals in the comparison group include patients with an ICD-10 diagnosis corresponding to opioid use disorder who were admitted to the three hospitals in the health system that did not implement an addiction consult service at any point during the study period. Individuals in the treatment group reflected patients with opioid use disorder treated at the hospital that implemented an addiction consult service at any point during the study period. MOUD=medication for opioid use disorder.

**Table 2: Sensitivity analysis with exclusion of a comparison hospital**

|  | Unadjusted | Adjusted |
| --- | --- | --- |
| Readmission within 30 days of discharge  (percentage points) | -0.02  [-0.07,0.03] | -0.01  [-0.05,0.04] |
| Length of stay  (days) | 0.24  [-0.65,1.12] | 0.25  [-0.60,1.10] |
| MOUD receipt  (percentage points) | 0.08^*^  [0.02,0.14] | 0.05*  [0.00,0.10] |
| Discharge on therapeutic MOUD  (percentage points) | 0.12^***^  [0.08,0.16] | 0.10^***^  [0.06,0.14] |
| Discharge before medically advised  (percentage points) | 0.01  [-0.03,0.06] | 0  [-0.04,0.04] |
| Emergency department visit within 6 months of discharge  (visits) | 0.02  [-0.03,0.07] | 0.03  [-0.01,0.08] |

Notes: This table depicts regression estimates that correspond to differential changes in outcomes between patients with OUD admitted to the hospital with an ACS (“treatment”) relative to patients with OUD admitted to the other hospitals without ACS (“comparison”), before and after the ACS was implemented in July 2023. All models include hospital- and year-fixed effects. Adjusted models additionally controlled for age, sex, race, insurance type, Charlson Comorbidity Index score, and the presence of serious injection-related infections. 95% confidence intervals are presented in brackets.

*p-value<0.05

***p-value<0.001

**Table 3: Sensitivity analysis with exclusion of March-December 2020 for COVID-19 pandemic**

|  | Unadjusted | Adjusted |
| --- | --- | --- |
| Readmission within 30 days of discharge  (percentage points) | -0.01  [-0.05,0.03] | 0  [-0.04,0.04] |
| Length of stay  (days) | 0.33  [-0.58,1.23] | 0.30  [-0.57,1.17] |
| MOUD receipt  (percentage points) | 0.05*  [0.00,0.12] | 0.03  [-0.01,0.07] |
| Discharge on therapeutic MOUD  (percentage points) | 0.09^***^  [0.05,0.14] | 0.08^***^  [0.04,0.12] |
| Discharge before medically advised  (percentage points) | 0  [-0.04,0.04] | -0.01  [-0.05,0.03] |
| Emergency department visit within 6 months of discharge  (visits) | 0.02  [-0.03,0.06] | 0.03  [-0.01,0.07] |

Notes: This table depicts regression estimates that correspond to differential changes in outcomes between patients with OUD admitted to the hospital with an ACS (“treatment”) relative to patients with OUD admitted to the other hospitals without ACS (“comparison”), before and after the ACS was implemented in July 2023. All models include hospital- and year-fixed effects. Adjusted models additionally controlled for age, sex, race, insurance type, Charlson Comorbidity Index score, and the presence of serious injection-related infections. 95% confidence intervals are presented in brackets.

*p-value<0.05

***p-value<0.001
